# Acacia Gum as a Natural Anti-Plasticizer for the Production of Date Syrup Powder: Sorption Isotherms, Physicochemical Properties, and Data Modeling

**DOI:** 10.3390/foods9010050

**Published:** 2020-01-05

**Authors:** Nasim Mansoori, Mahsa Majzoobi, Mohsen Gavahian, Fojan Badii, Asgar Farahnaky

**Affiliations:** 1 Department of Food Science and Technology, School of Agriculture, Shiraz University, Shiraz 7144165186, Iranasgar.farahnaky@rmit.edu.au (A.F.); 2 Biosciences and Food Technology, School of Science, RMIT University, Bundoora West Campus, Melbourne 3083, Australia; 3 Product and Process Research Center, Food Industry Research and Development Institute, No. 331 ShihPin Rd., Hsinchu 30062, Taiwan; 4 Agricultural Engineering Research Institute (AERI), Agricultural Research, Education and Extension Organization (AREEO), Karaj 31585-845, Iran

**Keywords:** acacia gum, anti-plasticizer, date syrup, date powder, modeling, powder stability, palm date, physicochemical properties, *Phoenix dactylifera*, sorption isotherm

## Abstract

The thermoplastic and hygroscopic behaviors of date syrup (DS) challenge the DS drying process. In this context, DS was mixed with 30%, 40%, 50%, and 60% acacia gum (AG) and subjected to a drum dryer. The chemical composition, bulk density (*p*_b_), caking degree (CD), glass transition temperature (Tg), and color values of DS powders were studied. The sorption isotherms were also obtained and compared to that of those predicted by mathematical models. According to the results, increasing the AG concentration enhanced the moisture content, *p*_b_, brightness, and Tg while it reduced the CD and equilibrium moisture sorption. All DS powders had type III isotherm behavior, i.e., similar to high-sugar foods. Guggenheim-Anderson-de Boer (GAB) and Peleg models were found to be suitable for fitting the experimental data and these models explained the monolayer moisture content decrease with increasing AG concentration. These results of the present study, for the first time, verified that the AG can be used as a natural anti-plasticizer agent for DS powder production.

## 1. Introduction

The fruit of the palm tree (*Phoenix dactylifera*) has played a crucial role in providing energy and nutritional components, such as dietary fibers, minerals (selenium, copper, potassium, and magnesium), antioxidants (carotenoids and phenolics), and vitamins B and C, for humans during the past millennia [1,2]. The high-quality date fruit is consumed when it is fresh and can be stored for several weeks under controlled storage conditions [3]. Besides, about 50% of the harvested dates are considered as surplus dates and cannot be used fresh [4]. Therefore, the utilization of surplus dates can maintain sustainable date cultivation, reduce food waste, and result in economic benefits for palm farmers and the food industry.

Date syrup (DS) powder could be produced from surplus dates as a healthier alternative to refined sugar considering its health benefits, including anti-inflammatory, anticancer, and anti-mutagenic effects [5]; the chemical composition, such as high sucrose content [6]; and the relatively low price of the surplus dates [4]. However, it was reported that DS is very difficult to dry due to its thermoplastic and hygroscopic behavior [7]. It also contains high concentrations of fructose (31%) and glucose (30%) [8] with glass transition temperatures (Tg) of −5 and 32 °C, respectively [9,10]. Anti-plasticizers and hydrocolloids, such as starch and acacia gum (AG), have been utilized as support materials to enhance the Tg of sugar-rich foods during the dehydration process [7]. Anticaking agents are also commonly used to improve the resulted powder flowability [9,11]. Combinations of AG, modified starch, maltodextrin, and anticaking agents (e.g., tricalcium phosphate, and glycerol monostearate) have also been proposed to improve the drying process of high-sugar syrups [9,12].

AG is the exudate from the acacia tree with stabilizing, emulsifying, and thickening properties [13,14,15,16]. This Codex Alimentarius-listed hydrocolloid [16] has been used as a drying agent in honey powder production and resulted in a product with low moisture content, good flowability, and physical properties [17]. Besides, good progress has been made in drying date fruit [18]. Also, in a previous study of our research team, the applicability of maltodextrin for date syrup drying was explored, which revealed the possibility of the production of date syrup powder by incorporating anti-plasticizers [19]. However, to the best of our knowledge, there is no published data on the potential application of AG as a processing aid in the production of DS powder. Thus, the current work aimed to investigate the effects of AG on the sorption isotherm and physicochemical properties of drum-dried DS and to determine the optimum concentration of this hydrocolloid, which results in a DS powder with enhanced physicochemical properties.

## 2. Materials and Methods

### 2.1. Chemicals and Date Syrup

The commercially produced syrup from the Kabkab date variety was provided by a local supplier in the south of Iran (Bushehr, Iran). According to the information provided by the supplier, the DS production includes date rehydrating, heating, mixing/crushing, separating the pulp from pits, and concentrating the product. The AG was supplied by a local supplier (Shojaiee Co., Shiraz, Iran). All the chemicals used in this work were of analytical grade and obtained from Merck Company (Darmstadt, Germany) unless otherwise specified.

### 2.2. Preliminary Studies

A set of preliminary tests were performed to figure out the appropriate range of AG concentration and the mixing conditions of syrup and gum. The minimum possible concentration of food additives (e.g. synthetic anti-plasticizer and anticaking agents) is usually preferred as they may negatively affect the product’s sensory properties (e.g., flavor and color) [20]. On the other hand, an insufficient concentration of anti-plasticizer causes difficulties in scrapping off the dehydrated product from the drum dryer surface and results in a sticky powder that is difficult to process and handle [19]. To define the appropriate range of AG concentration, a wide range of this hydrocolloid (5%, 10%, 20%, 30%, 40%, 50%, 60%, and 70%) were mixed with the date syrup, and the process of drying and the dried products were carefully investigated based on a preliminary investigation. According to the results, a minimum of 30% of AG was required to produce a dry powder. In addition, the incorporation of 70% AG in the formulation resulted in a product with unpleasant sensory properties. Therefore, the concentration of AG in the main experimental design ranged from 30% to 60%. To find the appropriate mixing conditions, five minutes [19] of either mild (at ambient temperature) or warm (at 80 °C) mixing before drum drying were evaluated. Afterward, the hot mixing condition was selected as the preferred method due to the more homogenous structure of the DS-AG mixture.

### 2.3. Sample Preparation

The mixture of DS and AG was mixed thoroughly for 5 min (MKg magnetic stirrer, Wertheim, Germany) while heating to 80 °C. DS powders were produced from mixtures that contained four DS to AG ratios, i.e., 70:30, 60:40, 50:50, and 40:60 (dry basis). These mixtures were carefully poured over a roller dryer (Reliance, Frisco, TX, USA) with a diameter of 42 cm and a rotation speed of 4 rpm. This drier was preheated through steam circulation at the pressure of 2.3 bars and had a surface temperature of about 130 °C. The resulted dried film was surface scrapped, cooled down, and milled by a hammer mill. The resulting product was collected instantly and sealed in airtight polythene plastic bags to prevent the powders from absorbing the moisture of the environment. These plastic pouches were kept at room temperature before further analysis.

### 2.4. Chemical Composition

The protein, fat, ash, and dietary fiber content of the DS and AG were assessed according to the suggested methods by the Association of Official Analytical Chemists (AOAC) (AOAC, 2000). For moisture content determination, three grams of each sample were dried at 70 °C until constant weight by a laboratory oven [19]. To determine the ash content, about three grams of each sample were combusted in a muffle furnace (C-1500, Asangodaz, Iran) at 550 °C for eight hours. The Kjeldahl method was employed to measure the amount of nitrogen in the sample, which was then converted to the protein content using the conversion factor of 6.25. The amount of fat in samples was measured according to the weight loss of samples (2 g) after fat extraction by hexane in a Soxhlet apparatus (Fisher, Racine, WI, USA) at 60 °C for 8 h.

### 2.5. Sugar Analysis

Sugar molecules of the DS sample were analyzed using a Shimadzu high-performance liquid chromatography (HPLC) (Shimadzu, Japan) along with a Shimadzu LC-9A liquid chromatograph pump (Shimadzu, Japan), and a RID-6A refractive index detector (Shimadzu, Japan) along with a data processing system (Nelson Analytical Inc., Paramus, NJ, USA). An aliquot (5 µL) of the filtered liquid was injected into an SCR-101N column of the HPLC system. A mixture of water and acetonitrile with a ratio of 20:80 and at a flow rate of 0.6 mL·min^−1^ was employed as the mobile phase to obtain high-resolution peaks of glucose, sucrose, and fructose. The runtime was 20 min and temperatures of both the detector and column were kept constant (60 °C). Standard solutions of glucose, sucrose, and fructose were used to prepare the calibration curves. The retention times of the peaks were compared with that of the standards injected at the same HPLC condition and quantified according to the regression equation, which was obtained from the standard samples [19].

### 2.6. Bulk Density

The ratio of the measured mass (kg) to the volume (m^3^) of the powder was defined as the bulk density (*p*_b_) of the date powders [21]. In this regard, a cylindrical shape vessel with a diameter of 2.6 × 10^−2^ m and a height of 4.1 × 10^−2^ m was filled with the date powder sample. The vessel was tapped 4 times and a ruler was slid across the edge of the cylinder to remove any excess amount of powders. An analytical balance was used to measure the mass (kg) of the date powder in the vessel. The vessel volume was calculated according to Equation (1):

V = πr^2^h, (1)

where V is the volume (m^3^), and “r” and “h” are the radius (m) and height (m) of the vessel, respectively.

### 2.7. Caking Degree

To determine the degree of caking (CD), one gram of each powder was put in an airtight glass jar (10 mL) containing a supersaturated solution of sodium iodide (NaI). Therefore, the relative humidity inside the glass jar was 33%. The jar was stored for a week at 25 °C and regularly weighed using a laboratory scale (H20/A, Mettler, Zurich, Switzerland) to make sure moisture equilibrium condition is available inside the jar [22]. The powders were put in an isotope (281A, Fisherbrand, Hilden, Germany) under vacuum condition and at 70 °C. The hydration process was continued to the time that successive weighing at 2-h intervals gave variation values of below 0.3%. The dehydrated powders were then weighed and transferred into a sieve with a mesh size of 500 μm, which was shaken for five minutes. The mass (kg) of the remaining powder on top of the sieve was measured and the *CD* was determined according to Equation (2) [9].

(2) $$CD\;=\;\frac{a}{b}\times 100,$$

where *CD* is the caking degree (%), and *b* and *a* are the total sample weight and the weight of the sample that remained on the sieve after the sieving process, respectively.

### 2.8. Glass Transition Temperature

The Tg of the prepared powders was assessed in triplicates using a differential scanning calorimeter (DSC) (Mettler, Switzerland) along with a cooling system (Admiral, East Brunswick, NJ, USA). Aluminum pans, which were completely airtight sealed, were utilized to prevent moisture loss while analyzing the samples. Briefly, 5 to 10 mg of each sample (572, Kern & Sohn GmbH, Balingen, Germany) were sealed in an aluminum pan and cooled to −40 °C. The temperature of the samples was kept constant for five minutes and then they were heated to achieve the target temperature of 150 °C at the constant heating rate of 10 °C.min^−1^. The samples were then equilibrated at 150 °C for five minutes and then cooled to −40 °C at the cooling rate of 10 °C.min^−1^. Re-scans were immediately carried out to confirm the existence of the Tg. A clean aluminum pan without any sample was used as the reference. The DSC thermograms were assessed for the enthalpy of melting and the onset, mid, and end of the transition [19].

### 2.9. Color Evaluation

Color values (L: Lightness; a: Redness-greenness; and b: Yellowness-blueness) of the date powders were analyzed using digital imaging and image analysis using the Photoshop software V.8 (Adobe Inc., Aurora, IL, USA) in the “Lab” mode [23,24]. Briefly, a digital DSC-W570 camera (Sony Corporation, Tokyo, Japan) was placed at a 0.3-m distance from the powder surface in a white box (0.5 × 0.5 × 0.6 m) along with a 40 W fluorescent lamp. The angle between the sample surface and the lamp was 45° while the angle between the axis of the camera lens and the sample surface was 90°. The digital photos were analyzed by Photoshop software to obtain the color parameters [24].

### 2.10. Determination of Isotherms

A range of a_w_ from 0.095 to about 0.934 (at specified temperatures) was prepared using six supersaturated salt solutions. The water activity (a_w_) of these solutions at 60, 40, 25, and 5 °C was obtained from the literature [25] (Table 1). Afterward, 2.0 g of each powder sample were weighed (572, Kern & Sohn GmbH, Balingen, Germany) and put in hermetically sealed polyethylene vessels containing supersaturated salt solutions. These vessels were stored in laboratory ovens (805 General Electric, New York, NY, USA) at 60 ± 0.1, 40 ± 0.1, 25 ± 0.1, and 5 ± 0.1 °C. The sample weight was monitored using a digital milligram precision scale first on the third day of storage followed by one-day intervals measurement. This test was continued to the time that no change was observed in the weight of the samples, i.e., 5 to 15 days (depending on the temperature and the sample formulation). The equilibrium moisture of the powders was reported as the mass ratio of water molecules to dry solid.

### 2.11. Data Modeling

Three well-known empirical models for correlating the relative humidity of the environment to the equilibrium moisture contents (EMCs) of samples, namely Brunauer–Emmett–Telle (BET), Guggenheim, Anderson, and de Boer (GAB), and Peleg models (Equations (3)–(5)), were used to fit the experimental data and determine the amount of monolayer water (monolayer moisture value) of the DS powders [19]. The water activity-EMC findings of all six water activity data points were employed for fitting to all models (i.e., GAB, Peleg, and BET). In this regard, the “Solver” function of the Microsoft Excel application (Microsoft Office, 2007, USA) was used [26] to minimize the residual sum of squares (RSS) between the experimental (exp) and predicted (pre) results. Several statistical parameters (Equations (6)–(9)), including the correlation coefficient (R^2^), standard error of estimate (SEE), mean relative percentage deviation modulus (M_e_), and RSS, were employed to assess the equations’ suitability. The R^2^ was also calculated to estimate the proportion of variability correlated with each model. The SEE represents the precision of the experiment estimation. The equations that had the minimum values of SEE and RSS and the greatest value of R^2^ were regarded as the most appropriate [27]. It was previously illustrated that M_e_ values lower than 10% indicate an appropriate fit of the mathematical model to the experimental data [28]:

(3) $$EMC\;=\;\frac{M_{0}.C.\;a_{w}.K}{\left(1-K.a_{w}\right)\left(1-K.a_{w}+C.K.a_{w}\right)},$$

(4) $$\mathrm{EMC}\;=\;\;\frac{M_{0}.C.a_{w}}{(1-a_{w)\left(1-C.a_{w}-a_{w}\right)}},$$

(5) $$EMC\;=\;k_{1}a_{w}^{n1}+\;k_{2}a_{w}^{n2},$$

where *EMC* and *M*_0_ are the equilibrium and monolayer moisture contents (% dry basis), respectively. “*a_w_*” is the water activity and *C*, *K*, *k*_1_, *k*_2_, *n*_1_, and *n*_2_ are model constants:

(6) $$\mathrm{RSS}\;=\;\sum_{i=1}^{n}{\left(Mi,exp-Mi,pre\right)}^{2\;\;},$$

(7) $$\mathrm{SEE}\;=\;\sqrt{\sum_{i=1}^{n}\frac{{\left(Mi,exp-Mi,pre\right)}^{2}}{n}},$$

(8) $$M_{e}\;=\;\frac{100}{n}\sum_{i=1}^{n}\frac{|Mi,exp-Mi,pre|}{Mi,exp}.$$

### 2.12. Statistical Analysis

All the tests were performed in triplicates and SPSS 16.0 (SPSS Inc., Chicago, IL, USA) was employed to analyze the data. Duncan’s multiple range test (*p* < 0.05) was utilized to detect significant differences between the results.

## 3. Results and Discussion

### 3.1. Chemical Properties of the Date Syrup

The chemical composition of the DS sample is presented in Table 2. According to the results, the concentrations of polysaccharides (glucose, fructose, and sucrose), protein, and fat in the studied DS were 74.5%, 0.8%, and 0.1%, respectively. In addition, the chemical analysis revealed that this DS has about 1% sucrose (molecular weight of 342 mol/g), 30% glucose (molecular weight of 180 mol/g), and 34% fructose (molecular weight of 180 mol/g). These carbohydrate molecules affect the Tg of food products [29]. Therefore, it is important to understand the concentration of these molecules in a product that is supposed to be dried. According to the literature, the total sugar content of DS may reach about 88%, and fructose and glucose are the two main sugars molecules of this syrup [30]. Besides, DS contains some other elements, including minerals, metal ions, and vitamins, which can make it a nutritious food commodity. In the present study, the ash content of the DS was approximately 2%, including non-fat, non-sugar, and non-protein components (Table 2). Similar to the DS, the AG used in the present study contained limited amounts of fat, protein, and ash (2.5%, 0.14%, and 3.11%, respectively).

### 3.2. Physical Properties of the Date Syrup Powders

The preliminary results revealed that the production of DS powder without incorporation of AG was not feasible due to the high CD of the dehydrated DS samples. Physical properties of the DS powder samples (containing 30%, 40%, 50%, and 60% AG), including color values, *p*_b_, CD, and Tg, are presented in Table 3. According to the results, the *p*_b_ of the samples increased from 590 to 610, 690, and 730 kg·m^−3^ when the concentration of AG was increased from 30% to 40%, 50%, and 60%, respectively. This observation is related to the higher *p*_b_ of AG (730 kg·m^−3^) than that of the pure DS powder. These data indicate that the *p*_b_ of DS powders increased with the AG level. Moreover, the addition of AG to DS reduced the CD and hence can enhance the storage and handling ability of the resulting powder. The decreased CD could be correlated with the Tg. According to Table 3, the Tg of the DS was −1.27 °C, which indicates that this food commodity is expected to be at its rubbery state when stored at room temperature. As the AG has a higher Tg (84.73 °C) than DS (−1.27 °C), it acts as an anti-plasticizer component by increasing the Tg of the mixture. Therefore, the Tg of the syrup increased with increasing the AG concentration. The Tg values of the DS powders with 30%, 40%, 50%, and 60% AG levels were 10.2, 17.4, 28.2, and 40.9 °C, respectively. Consequently, increasing the concentration of AG from 0% to 60% greatly decreased the CD from 54.82% to 0.08%.

### 3.3. Sorption Isotherms

The EMC of samples at the studied temperatures are shown in Table 4. The EMC of AG-containing samples (DS-AG) was less than that of DS at similar a_w_ values. This observation could be related to the ability of AG in reducing the hygroscopic properties of mixtures [31]. The incorporation of this hydrocolloid in DS changed the hydrophilic–hydrophobic balance and affected the amount of adsorbed H_2_O molecules. Furthermore, the water sorption phenomenon can be related to the swelling-caused changes in the polymer structural [32]. According to the literature, the stability of AG-containing systems might be correlated with the a_w_ and Tg, which were affected by gum incorporation [33]. Therefore, the samples were sticky at temperatures above Tg and non-sticky at temperatures below Tg. Likewise, a study on the impacts of AG on Tg of strawberry pulp powder showed that increasing the hydrocolloid concentration enhanced the Tg of the mixture [12]. It also showed that the EMC of AG-containing samples was lower than the samples that were free from the gum. A similar observation was made for a dried mixture of pineapple syrup and AG [31]. Furthermore, researchers confirmed that enhancing the moisture content (from 0% to 30%) reduced the Tg of lyophilized pineapple pulp whereas the addition of AG minimized this highly plasticizing effect of water [31].

**Figure 1:**
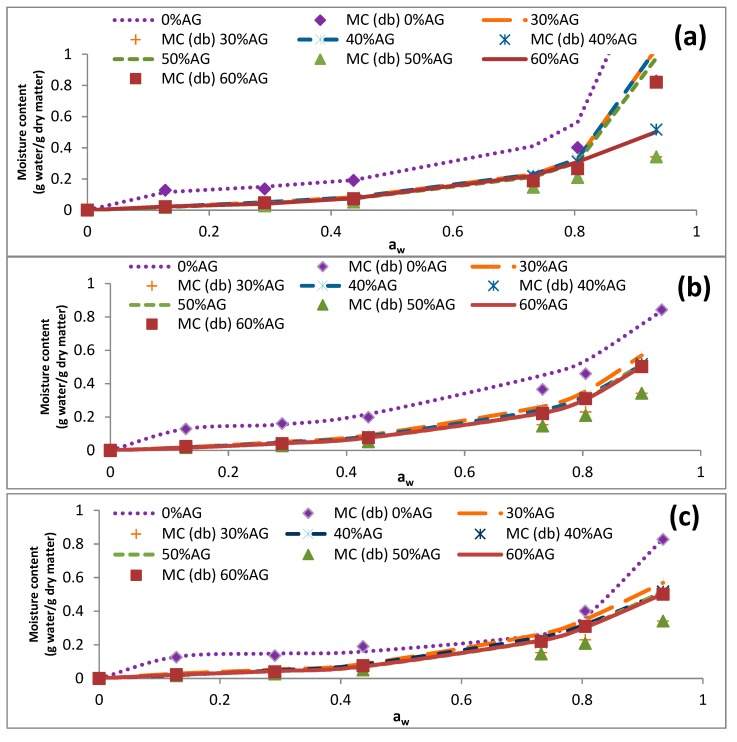
Effects of acacia gum concentration on the equilibrium moisture sorption isotherm of date syrup powders at 5 °C. The experimental data are presented as symbols and the data obtained by fitting the experimental data into mathematical models are presented as lines. (**a**–**c**) represent the obtained data from BET, GAB, and Peleg models. 0% AG: 100% DS-0% AG; 30% AG: 70% DS-30% AG; 40% AG: 60% DS-40% acacia gum; 50% AG: 50% DS-50% AG; 60% AG: 40% DS-60% acacia gum; DS: date syrup; AG: acacia gum.

**Figure 2:**
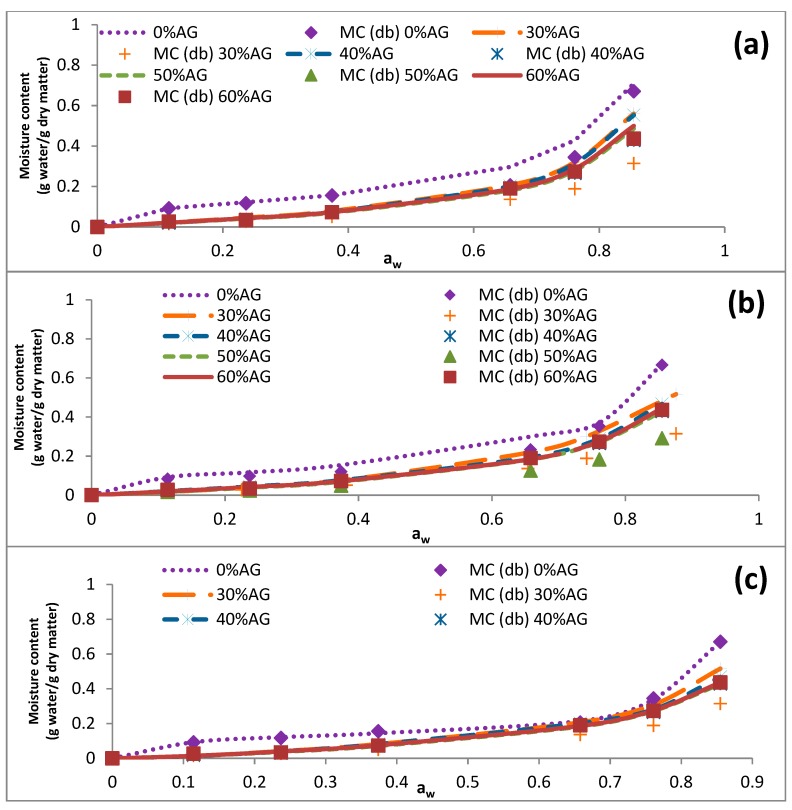
Effects of acacia gum concentration on the equilibrium moisture sorption isotherm of date syrup powders at 25 °C. The experimental data are presented as symbols and the data obtained by fitting the experimental data into mathematical models are presented as lines. (**a**–**c**) represent the obtained data from BET, GAB, and Peleg models. 0% AG: 100% DS-0% AG; 30% AG: 70% DS-30% AG; 40% AG: 60% DS-40% acacia gum; 50% AG: 50% DS-50% AG; 60% AG: 40% DS-60% acacia gum; DS: date syrup; AG: acacia gum.

**Figure 3:**
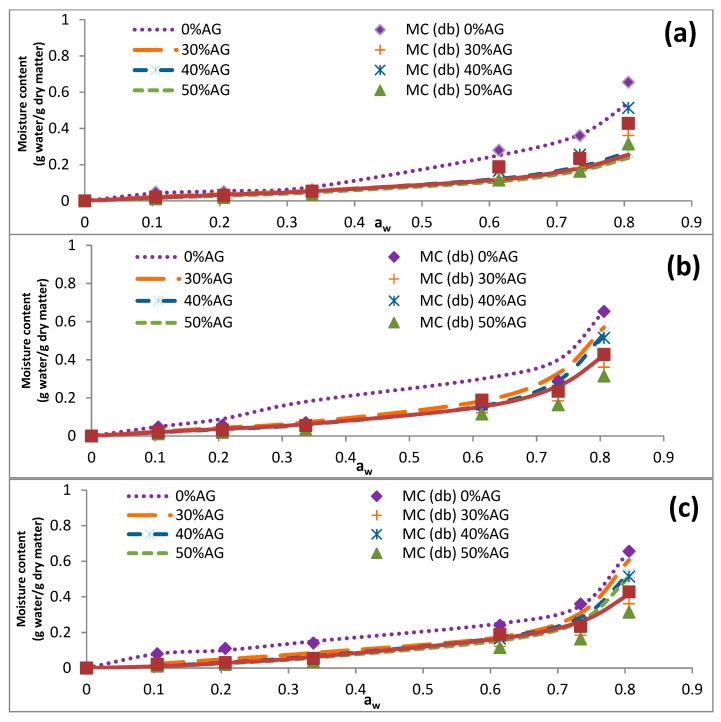
Effects of acacia gum concentration on the equilibrium moisture sorption isotherm of date syrup powders at 40 °C. The experimental data are presented as symbols and the data obtained by fitting experimental data into mathematical models are presented as lines. (**a**–**c**) represent the obtained data from BET, GAB, and Peleg models. 0% AG: 100% DS-0% AG; 30% AG: 70% DS-30% AG; 40% AG: 60% DS-40% acacia gum; 50% AG: 50% DS-50% AG; 60% AG: 40% DS-60% acacia gum; DS: date syrup; AG: acacia gum.

**Figure 4:**
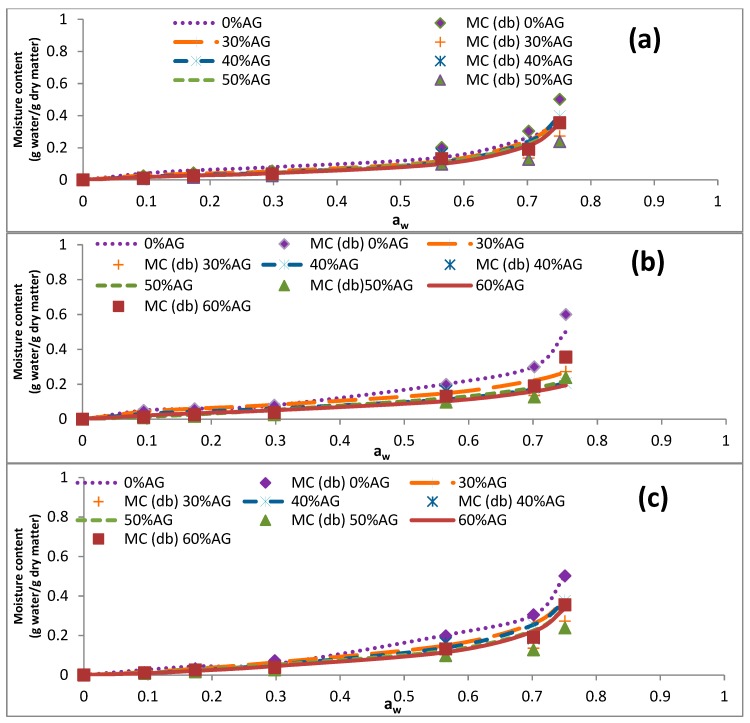
The effect of acacia gum concentration on the equilibrium moisture sorption isotherm of date syrup powders at 60 °C. The experimental data are presented as symbols and the data obtained by fitting the experimental data into mathematical models are presented as lines. (**a**–**c**) represent the obtained data from BET, GAB and Peleg models. 0% AG: 100% DS-0% AG; 30% AG: 70% DS-30% AG; 40% AG: 60% DS-40% acacia gum; 50% AG: 50% DS-50% AG; 60% AG: 40% DS-60% acacia gum; DS: date syrup; AG: acacia gum.

The data presented in Table 4 illustrates that the EMC of samples was reduced by an increase in the temperature (5–60 °C). Similar results were reported previously for cowpea and fig samples [34,35]. At elevated temperatures, intermolecular forces of attraction diminish because of the increased kinetic energy of molecules, resulting in increased molecular mobility. For that reason, H_2_O molecules, which have limited motions, are expected to form stronger bonds with the binding sites of polymer molecules when the temperature is low [35]. Figure 1, Figure 2, Figure 3 and Figure 4 represent the sorption isotherms obtained from the experimental data at different temperatures along with graphs acquired from the studied mathematical models. Generally, all isotherms showed a slight increase and a sharp increase in the EMC at the low ranges of a_w_ and at the intermediate ranges of a_w_ (~0.5), respectively. This trend was comparable to a common pattern that was previously reported for food materials with a high sugar content [19,36]. This is because of the principal influence of solvent-solute interactions on the dissolution of sugar molecules [12]. In addition, the isotherm of the DS-AG powder was significantly different from that of DS. This observation was in line with those reported for some fruit samples in the literature [12,31]. According to the results, all the experimental isotherms in the present study are similar to that of the type III isotherm based on the classification of Brunauer [31].

The results showed that the anti-plasticizing effect of AG was different from those of maltodextrin for date powder production [19]. For example, at 5 °C (the a_w_ of 0.128), the EMC of samples was changed from 0.1269 to 0.0230 and 0.02113 when 60% of AG and maltodextrin was used in the formulation, respectively. In other words, incorporation of 60% AG and maltodextrin resulted in an 82% and 83% decrease in EMC (Table 4). Similarly, at 60 °C (a_w_ of 0.095), the EMC of the sample was changed from 0.0256 to 0.0116 and 0.0135 when 60% of AG and maltodextrin was used in the formulation, respectively; that is to say, incorporation of 60% AG and maltodextrin resulted in a 55% and 47% decrease in EMC of the samples, respectively. Indeed, the differences between the anti-plasticizing effects of these natural anti-plasticizers were more profound at higher temperatures. According to the data reported in the present study and a previous effort on date powder production [19], it can be highlighted that both AG and maltodextrin can be used as effective anti-plasticizers while the variations in the intensity of their effects suggest a need for an optimization study, in terms of concentration and type of anti-plasticizer, before commercial production of date syrup powder.

### 3.4. Data Modeling

The estimated constants of the studied equations, i.e., GAB, BET, and Peleg, are presented in Table 5.

Besides, the criteria for considering a mathematical equation as an appropriate model, including R^2^, RSS, SEE, and Me, are illustrated in Table 6. The greatest R^2^ values and the minimum RSS, SEE, and M_e_ values can be regarded as a sign of better correlations between the experimental and calculated data. According to the above-described criteria, GAB and Peleg models (R^2^ > 0.98) are suitable for the prediction of EMC of DS and DS-AG samples. According to the presented data in Table 6, the average M_e_ values of the Peleg and GAB model are considerably (about 90%) smaller than that of the BET model, which indicates that the experimental data can be satisfyingly fitted into these models.

The GAB model yielded average M_e_ values of 8.46% (ranging from 7.00% to 9.22%), 8.03% (ranging from 5.89% to 10.22%), 8.82% (ranging from 8.06% to 9.84%), 7.48% (ranging from 6.08% to 8.60), and 7.79% (ranging from 5.66% to 8.89%) for DS powders containing 0% (control sample), 30%, 40%, 50%, and 60% AG, respectively. Furthermore, Peleg models yielded average M_e_ values of 8.07%, 7.98%, 7.71%, 6.74%, and 3.8% for DS powders containing 0%, 30%, 40%, 50%, and 60% AG, respectively. Moreover, average M_e_ values of 15.38%, 17.23%, 16.33%, 19.04%, and 19.21% were obtained from the BET model for DS powders containing 0%, 30%, 40%, 50%, and 60% AG, respectively. These findings were in agreement with that documented by Aviara et al. (2006), who mentioned that only relying on RSS and SEE is an inappropriate approach to determine the suitability of a model for fitting experimental moisture sorption isotherms [37]. It seems that the GAB and Peleg equations gave the most appropriate fit for the sorption isotherms of the studied samples with a wide range of a_w_ according to the SEE, RSS R^2^, and M_e_ values (Table 6). This also indicates that the BET model was not able to describe the water activity-EMC well in DS powder, indicating the phenomena of dissolution may predominate over sorption. Therefore, concentrations of the monolayer water (M_0_) at various temperatures were obtained through these two models. The M_0_ values of the DS samples were higher than those of the AG-containing samples. Higher concentrations of AG resulted in smaller amounts of M_0_. This was the case for both the GAB and BET models at all the studied temperatures. AG has the role of an effective anti-plasticizer in the present study. This anti-plasticizer has a larger size and a higher molecular weight than small sugar molecules of DS. These characteristics of AG can decrease the monolayer water level by reducing the overall ratio of interaction sites.

According to the results, M_0_ values of the control (DS) at 5 to 60 °C varied from 0.06 to 0.11 and from 0.06 to 0.37 (g water/g dry solids) for the BET and GAB models, respectively. Likewise, a previously conducted study calculated 0.06 to 0.17 (g water/g dry solids) for M_0_ values of date paste at 5 to 40 °C [38]. The results of the current study revealed that the M_0_ value was reduced by an increase in the temperature (from 5 to 60 °C). A similar observation was reported for fig, pineapple, and DS [31,35,38]. The reduction of M_0_ may be illustrated by highlighting the effects of temperature on the physicochemical characteristics of the biopolymers. The strength of hydrogen bonds of polymers can be reduced at elevated temperatures. This phenomenon can enhance the availability of active sites of the polymer for binding water molecules, which can increase the amount of monolayer water [35].

According to the literature, reduced temperatures can facilitate the formation of powerful exothermic adsorbent–adsorbate interactions, enhancing the C parameter of Equation (1) [31]. A similar observation was made in the current study. According to the results, increasing the temperature from 5 to 60 °C enhanced the C parameter steadily (Equations (3) and (4)). Likewise, a non-regular variation of the C parameter due to temperature variations was observed previously for immature acerola juice powder containing AG.

The K parameter, which represents the interactions between multilayer molecules and the adsorbent, is prone to range from the energy level of the molecules in liquid water to that of the monolayer. The water multilayers are expected to have liquid water properties when the K value is approximately one [32]. Similarly, in the current study, the K value of DS powders showed only negligible changes (ranged from 0.8 to 1.2).

## 4. Conclusions

This paper described the first attempt to improve the physical stability of date syrup through the incorporation of a natural hydrocolloid, i.e., acacia gum. The results showed that this gum can be successfully incorporated into date syrup as an anti-plasticizer to make the production of date syrup powder feasible. Moreover, it was revealed that increasing the acacia gum concentration (from 30% to 40%, 50%, and 60%) enhanced the moisture content, *p*_b_, lightness, and Tg of the date syrup powder. On the other hand, increasing the gum concentration produced date syrups with reduced equilibrium moisture and caking degree. For all the DS powders, type III isotherm behavior was observed, which was similar to that of high-sugar food materials. Furthermore, this study revealed that Peleg and GAB are appropriate models to fit the experimental data of date syrup acacia gum mixtures. Fitting the experimental data into these models revealed that increasing the acacia gum concentration decreased the monolayer moisture content. According to the findings of this study, food and pharmaceutical industries may consider acacia gum as an effective natural anti-plasticizer to produce a date syrup powder with improved storage properties and reduced caking degree. Besides, replacing chemically produced anti-plasticizers with natural alternatives, such as acacia gum, can improve the market competitiveness of the dried date powder producers through complying with the clean- and green-label policy as well as consumer food trends.

## Abbreviations and Nomenclatures

°Bxdegrees Brix0% AGdate syrup with no acacia gum30% AGpowder samples containing 70% date syrup and 30% acacia gum40% AGpowder samples containing 60% date syrup and 40% acacia gum50% AGpowder samples containing 50% date syrup and 50% acacia gum60% AGpowder samples containing 40% date Syrup and 60% acaciaAOACAssociation of Official Analytical Chemists AGacacia guma_w_water activityCa constant in GAB and BET modelsCDcaking degreeDSdate syrupDS-AGdate syrup- acacia gum mixturesEMCequilibrium moisture contentexpexperimentalGABGuggenheim-Anderson-de BoerHthe height of the vessel used for bulk density analysisHPLChigh-performance liquid chromatographyIUPACInternational Union of Pure and Applied ChemistryKa constant in GAB modelk_1_a Peleg model constantk_2_a Peleg model constantM_0_monolayer moisture contentM_e_mean relative percentage deviation modulusn_1_a Peleg model constantn_2_a Peleg model constant
*p*_b_
bulk densityPrepredictedRradius of the vessel used for bulk density analysisR^2^correlation coefficientRSSresidual sum of squaresSEEstandard error of estimateTemp.temperatureTgglass transition temperatureTgcendset glass transitionTgionset glass transitionTgmmiddle glass transitionVvolume of the bulk density measurement vessel

## Figures and Tables

**Table 1:** The a_w_ values of saturated salt solutions at various temperatures.

Type of Saturated Salt Solution	Temperature (°C)			
(IUPAC ID/Chemical Formula)	5	25	40	60
Lithium chloride (LiCl)	0.128	0.114	0.105	0.095
Potassium acetate (CH_3_COOK)	0.291	0.237	0.206	0.174
Sodium iodide (NaI)	0.437	0.374	0.337	0.298
Sodium nitrite (NaNO_2_)	0.732	0.658	0.614	0.565
Sodium chloride (NaCl)	0.805	0.761	0.734	0.702
Potassium chloride (KCl)	0.934	0.855	0.806	0.751

**Table 2:** Physicochemical properties of acacia gum and date syrup used in the present study.

Component	DS *	AG
Moisture content (% *w*/*w*)	14.42 ± 1.95 ^a^	9.17 ± 0.19 ^b^
Protein (% *w*/*w*)	0.76 ± 0.06 ^b^	2.50 ± 1.07 ^a^
Fat (% *w*/*w*)	0.10 ± 0.00 ^a^	0.14 ± 0.01 ^a^
Ash (% *w*/*w*)	2.23 ± 0.17 ^b^	3.11 ± 0.17 ^a^
Glucose (% *w*/*w*)	39.63 ± 0.08 ^a^	NA ^b^
Fructose (% *w*/*w*)	33.68 ± 0.14 ^a^	NA ^b^
Sucrose (% *w*/*w*)	1.25 ± 0.00 ^a^	NA ^b^
°Bx	82.00 ± 0.05 ^a^	NA ^b^
Total carbohydrates ** (% *w/w*)	~74.56	~85.08 **

* Data are presented as the mean value of three replicates. Values with different superscripts in each row are significantly different (*p* < 0.05). NA: not applicable; DS: Date syrup; °Bx: degree of brix; Ag: acacia gum. ** The total carbohydrate content of date syrup represents the summation of glucose, fructose and sucrose contents. This value for the acacia gum is based on the non-protein, non-ash and non-fat proportion of the gum, which mainly consists of D-galactose and L-arabinose according to the literature [13].

**Table 3:** Glass transition temperature (Tg), caking degree (CD), color parameters (L, a, and b), and density (*p*_b_), of samples with various acacia gum concentrations. 0% AG: 100% DS-0% AG; 30% AG: 70% DS-30% AG); 40% AG: 60% DS-40% acacia gum; 50% AG: 50% DS-50% AG; 60% AG: 40% DS-60% acacia gum; CD: caking degree; DS: date syrup; AG: acacia gum; Tg: glass transition; Tgi: onset; Tgm: mid-point; Tgc: endset.

Sample	*p*_b_ (kg·m^−3^)	Color Values *					Tg (°C)	
		L	a	b	CD (%)	Tgi	Tgm	Tgc
0% AG	-	11.66 ± 3.05 ^c^	7.33 ± 0.57 ^a^	3.33 ± 0.5 ^f^	-	−8.00 ± 188 ^f^	−1.27 ± 97 ^f^	5.19 ± 2.16 ^f^
30% AG	590 ± 1 ^c^	64.33 ± 0.57 ^b^	4.66 ± 0.57 ^a^	47.66 ± 1.52 ^a^	54.82 ± 0.23 ^a^	5.06 ± 0.21 ^e^	10.22 ± 0.16 ^e^	15.73 ± 0.33 ^e^
40% AG	610 ± 0 ^c^	66.00 ± 1.00 ^a,b^	4.66 ± 0.57 ^a^	44.00 ± 1.00 ^b^	3.11 ± 0.25 ^b^	9.94 ± 0.38 ^d^	17.43 ± 0.35 ^d^	25.81 ± 1.17 ^d^
50% AG	690 ± 0 ^b^	67.53 ± 1.00 ^a^	0.33 ± 0.57 ^b^	42.33 ± 0.57 ^c^	0.32 ± 0.03 ^c^	14.84 ± 0.06 ^c^	28.25 ± 0.21 ^c^	41.29 ± 0.34 ^c^
60% AG	730 ± 3 ^a^	67.66 ± 1.15 ^a^	0.66 ± 0.57 ^b^	36.33 ± 0.57 ^d^	0.08 ± 0.00 ^d^	28.09 ± 0.82 ^b^	40.87 ± 0.58 ^b^	52.88 ± 0.26 ^b^
AG	730 ± 1 ^a^	68.33 ± 0.57 ^a^	−9.00 ± 0.00 ^c^	17.33 ± 0.57 ^e^	0.00 ± 0.00 ^d^	76.90 ± 0.93 ^a^	84.73 ± 1.89 ^a^	91.47 ± 1.72 ^a^

* The presented data are the mean value of 3 replicates ± standard deviations. Values with different superscripts in each column are significantly different (*p* < 0.05).

**Table 4:** Equilibrium moisture content (EMC) of the studied samples at various temperatures. 0% AG: 100% DS-0% AG; 30% AG: 70% DS-30% AG; 40% AG: 60% DS-40% acacia gum; 50% AG: 50% DS-50% AG; 60% AG: 40% DS-60% acacia gum; DS: date syrup; AG: acacia gum.

Temperature (°C)	a_w_	EMC of Samples (g Water/g Dry Matter)				
		0% AG	30% AG	40% AG	50% AG	60% AG
5	0.128	0.127 ± 0.001	0.031 ± 0.002	0.022 ± 0.001	0.025 ± 0.002	0.023 ± 0.000
	0.291	0.136 ± 0.003	0.045 ± 0.000	0.040 ± 0.000	0.037 ± 0.002	0.040 ± 0.002
	0.437	0.192 ± 0.004	0.098 ± 0.000	0.093 ± 0.001	0.077 ± 0.002	0.076 ± 0.000
	0.732	0.353 ± 0.005	0.242 ± 0.015	0.228 ± 0.003	0.217 ± 0.009	0.220 ± 0.001
	0.805	0.402 ± 0.005	0.372 ± 0.010	0.337 ± 0.006	0.312 ± 0.009	0.310 ± 0.001
	0.934	0.828 ± 0.016	0.566 ± 0.009	0.560 ± 0.008	0.516 ± 0.003	0.502 ± 0.012
25	0.114	0.092 ± 0.006	0.023 ± 0.001	0.022 ± 0.003	0.021 ± 0.000	0.027 ± 0.00
	0.237	0.117 ± 0.011	0.037 ± 0.001	0.040 ± 0.000	0.031 ± 0.001	0.034 ± 0.002
	0.374	0.155 ± 0.004	0.081 ± 0.001	0.077 ± 0.001	0.078 ± 0.000	0.073 ± 0.001
	0.658	0.305 ± 0.011	0.209 ± 0.007	0.203 ± 0.002	0.188 ± 0.004	0.190 ± 0.002
	0.761	0.384 ± 0.005	0.308 ± 0.002	0.281 ± 0.002	0.267 ± 0.004	0.273 ± 0.002
	0.855	0.670 ± 0.010	0.516 ± 0.004	0.469 ± 0.008	0.429 ± 0.010	0.436 ± 0.008
40	0.105	0.049 ± 0.003	0.022 ± 0.001	0.016 ± 0.001	0.014 ± 0.004	0.021 ± 0.001
	0.206	0.052 ± 0.001	0.039 ± 0.000	0.030 ± 0.004	0.026 ± 0.001	0.031 ± 0.002
	0.337	0.062 ± 0.001	0.056 ± 0.000	0.0584 ± 0.001	0.054 ± 0.004	0.054 ± 0.001
	0.614	0.280 ± 0.006	0.234 ± 0.003	0.179 ± 0.002	0.163 ± 0.007	0.188 ± 0.002
	0.734	0.360 ± 0.006	0.293 ± 0.004	0.275 ± 0.003	0.255 ± 0.002	0.234 ± 0.004
	0.806	0.655 ± 0.008	0.577 ± 0.012	0.537 ± 0.009	0.513 ± 0.003	0.428 ± 0.005
60	0.095	0.026 ± 0.000	0.021 ± 0.001	0.010 ± 0.001	0.011 ± 0.000	0.012 ± 0.001
	0.174	0.044 ± 0.005	0.034 ± 0.000	0.032 ± 0.001	0.018 ± 0.001	0.026 ± 0.004
	0.298	0.056 ± 0.003	0.047 ± 0.002	0.037 ± 0.001	0.037 ± 0.000	0.0384 ± 0.001
	0.565	0.202 ± 0.004	0.186 ± 0.004	0.166 ± 0.005	0.161 ± 0.000	0.132 ± 0.001
	0.702	0.304 ± 0.006	0.193 ± 0.002	0.199 ± 0.008	0.185 ± 0.002	0.191 ± 0.008
	0.751	0.502 ± 0.003	0.409 ± 0.011	0.408 ± 0.004	0.356 ± 0.005	0.355 ± 0.005

**Table 5:** The calculated constants for the studied mathematical models (Peleg, GAB, and BET) and the corresponding monolayer moisture levels (M_0_) for the evaluated samples at various temperatures. 0% AG: 100% DS-0% AG; 30% AG: 70% DS-30% AG; 40% AG: 60% DS-40% acacia gum; 50% AG: 50% DS-50% AG; 60% AG: 40% DS-60% acacia gum; DS: date syrup; AG: acacia gum; M_0_: monolayer moisture content; C, K, k_1_, k_2_, n_1_ and n_2_: model constants.

Samples	Temperature (°C)	BET		GAB			Peleg			
		M_0_ (%)	C	M_0_ (%)	C	K	k_1_	k_2_	n_1_	n_2_
0% AG	5	11.1	53.1	37.54	0.29	0.82	1.14	0.18	8.26	0.16
30% AG	5	7.16	3.23	13.32	1.13	0.89	0.6	0.11	4.04	0.67
40% AG	5	7.01	2.70	12.92	1.07	0.88	0.39	1.64	3.1	0.95
50% AG	5	6.74	2.22	10.19	1.16	0.92	0.56	0.1	4.39	0.71
60% AG	5	6.61	1.69	10.85	1.12	0.91	0.53	0.1	4.23	0.76
0% AG	25	10.25	28.89	18.42	0.6	0.93	2.21	0.2	9.79	0.36
30% AG	25	7.54	2.75	9.62	1.84	0.97	1.22	0.35	10.5	1.48
40% AG	25	6.89	3.31	9.04	2.1	0.96	1.53	0.37	13.82	1.55
50% AG	25	6.18	1.99	9.66	1.59	0.94	1.00	0.33	11.39	1.55
60% AG	25	6.15	3.81	9.43	1.79	0.94	0.77	0.28	8.44	1.36
0% AG	40	9.27	2.34	8.80	0.52	1.1	7.42	0.28	13.45	1.19
30% AG	40	5.12	5.47	6.89	3.25	1.96	6.9	0.27	13.41	1.05
40% AG	40	5.65	3.60	5.70	3.22	1.11	6.61	0.22	13.37	1.04
50% AG	40	5.25	3.44	5.07	3.29	1.12	6.01	0.23	13.35	1.21
60% AG	40	5.08	4.29	6.11	3.25	1.06	3.2	0.32	13.45	1.46
0% AG	60	6.1	3.23	6.49	2.54	1.11	8.9	0.26	13.38	1.2
30% AG	60	4.21	8.13	4.06	11.78	1.19	7.89	0.29	13.43	1.26
40% AG	60	3.93	5.43	3.86	5.5	1.15	7.93	0.31	13.41	1.49
50% AG	60	4.80	2.50	4.18	4.33	1.17	7.8	0.25	13.42	1.32
60% AG	60	4.82	3.64	3.46	6.7	1.2	7.85	0.25	13.41	1.4

**Table 6:** The coefficient of determination (R^2^), residual sum of squares (RSS), standard error of estimate (SEE), and the mean relative percentage deviation moduli (Me) for the models applied to the experimental data of the studied sample at various temperatures. 0% AG: 100% DS-0% AG; 30% AG: 70% DS-30% AG; 40% AG: 60% DS-40% acacia gum; 50% AG: 50% DS-50% AG; 60% AG: 40% DS-60% acacia gum; DS: date syrup; AG: acacia gum.

		RSS				SEE				Me (%)					R^2^			
	Temperature (°C)	5	25	40	60	5	25	40	60	5	25	40	60	Overall%	5	25	40	60
Sample	Models																	
0% AG	BET	3.46 × 10^−2^	3.02 × 10^−2^	1.29 × 10^−2^	5.08 × 10^−2^	0.07030	0.02070	0.04280	0.02430	15.80	11.10	17.23	17.37	15.38	0.971	0.980	0.955	0.973
	GAB	1.46 × 10^−2^	2.47 × 10^−3^	1.36 × 10^−3^	7.08 × 10^−5^	0.04570	0.01877	0.01401	0.00318	8.66	9.22	7.00	8.97	8.46	0.988	0.992	0.995	0.999
	Peleg	4.59 × 10^−3^	2.50 × 10^−4^	1.33 × 10^−3^	4.08 × 10^−5^	0.02559	0.00597	0.01375	0.00241	7.31	8.63	7.75	8.60	8.07	0.989	0.999	0.995	0.999
30% AG	BET	5.00 × 10^−3^	1.00 × 10^−3^	5.92 × 10^−3^	3.30 × 10^−2^	0.0850	0.03820	0.02910	0.02910	18.27	17.25	18.79	14.60	17.23	0.980	0.975	0.962	0.983
	GAB	9.55 × 10^−4^	1.80 × 10^−4^	4.11 × 10^−3^	7.78 × 10^−3^	0.01168	0.00506	0.02423	0.00333	10.22	5.89	7.83	8.16	8.03	0.996	0.999	0.984	0.997
	Peleg	1.17 × 10^−4^	1.16 × 10^−4^	3.69 × 10^−3^	7.23 × 10^−3^	0.01292	0.00406	0.02350	0.00320	7.56	7.95	7.59	8.80	7.98	0.995	0.999	0.980	0.996
40% AG	BET	2.31 × 10^−3^	2.03 × 10^−3^	4.00 × 10^−3^	3.50 × 10^−3^	0.05550	0.01890	0.07801	0.02240	19.23	15.19	16.78	14.10	16.33	0.969	0.970	0.968	0.909
	GAB	5.05 × 10^−4^	1.96 × 10^−4^	7.44 × 10^−4^	5.33 × 10^−3^	0.00849	0.00528	0.00103	0.00270	8.06	8.76	8.61	9.84	8.82	0.997	0.998	0.996	0.996
	Peleg	4.36 × 10^−4^	1.07 × 10^−4^	2.49 × 10^−4^	5.27 × 10^−3^	0.00102	0.00390	0.00596	0.00274	7.51	7.06	7.82	8.46	7.71	0.998	0.999	0.998	0.998
50% AG	BET	1.22 × 10^−3^	9.34 × 10^−3^	1.00 × 10^−3^	3.00 × 10^−3^	0.03820	0.03732	0.04410	0.07002	19.25	18.10	19.11	19.69	19.04	0.972	0.980	0.970	0.967
	GAB	2.35 × 10^−4^	1.28 × 10^−4^	8.78 × 10^−4^	4.55 × 10^−4^	0.00579	0.00427	0.03526	0.02548	8.60	6.95	8.29	6.08	7.48	0.999	0.999	0.986	0.980
	Peleg	2.00 × 10^−4^	8.14 × 10^−5^	1.95 × 10^−4^	3.61 × 10^−4^	0.00535	0.00341	0.00527	0.02271	5.84	7.43	7.86	5.83	6.74	0.999	0.999	0.999	0.997
60% AG	BET	5.47 × 10^−3^	1.40 × 10^−3^	1.22 × 10^−3^	1.00 × 10^−3^	0.02713	0.04522	0.01401	0.0121	19.00	18.16	18.10	21.57	19.21	0.985	0.970	0.970	0.970
	GAB	8.44 × 10^−5^	1.82 × 10^−4^	2.12 × 10^−4^	1.68 × 10^−4^	0.0034	0.00510	0.00738	0.00551	5.66	8.89	8.33	8.29	7.79	0.999	0.998	0.985	0.983
	Peleg	9.90 × 10^−5^	2.16 × 10^−4^	1.61 × 10^−4^	1.58 × 10^−4^	0.00377	0.00555	0.00151	0.00150	3.32	2.11	4.80	3.28	3.38	0.999	0.998	0.989	0.984
